# FGF21 Protects against Aggravated Blood-Brain Barrier Disruption after Ischemic Focal Stroke in Diabetic db/db Male Mice via Cerebrovascular PPARγ Activation

**DOI:** 10.3390/ijms21030824

**Published:** 2020-01-28

**Authors:** Yinghua Jiang, Li Lin, Ning Liu, Qingzhi Wang, Jing Yuan, Yadan Li, Kelly K. Chung, Shuzhen Guo, Zhanyang Yu, Xiaoying Wang

**Affiliations:** 1 Clinical Neuroscience Research Center, Department of Neurosurgery, School of Medicine, Tulane University, New Orleans, LA 70112, USA; yjiang11@tulane.edu (Y.J.); nliu3@tulane.edu (N.L.); tmcnarror@126.com (Q.W.); yuanjing27@126.com (J.Y.); yli72@tulane.edu (Y.L.); 2 Neuroprotection Research Laboratory, Department of Radiology and Neurology, Massachusetts General Hospital, Harvard Medical School, Charlestown, MA 02129, USA; linliwz@163.com (L.L.); kkchung@mgh.harvard.edu (K.K.C.); sguo@mgh.harvard.edu (S.G.)

**Keywords:** focal ischemic stroke, type 2 diabetes, Db/db mouse, fibroblast growth factor 21, blood—brain barrier, human brain microvascular endothelial cell

## Abstract

Recombinant fibroblast growth factor 21 (rFGF21) has been shown to be potently beneficial for improving long-term neurological outcomes in type 2 diabetes mellitus (T2DM) stroke mice. Here, we tested the hypothesis that rFGF21 protects against poststroke blood–brain barrier (BBB) damage in T2DM mice via peroxisome proliferator-activated receptor gamma (PPARγ) activation in cerebral microvascular endothelium. We used the distal middle cerebral occlusion (dMCAO) model in T2DM mice as well as cultured human brain microvascular endothelial cells (HBMECs) subjected to hyperglycemic and inflammatory injury in the current study. We detected a significant reduction in PPARγ DNA-binding activity in the brain tissue and mRNA levels of BBB junctional proteins and PPARγ-targeting gene *CD36* and *FABP4* in cerebral microvasculature at 24 h after stroke. Ischemic stroke induced a massive BBB leakage two days after stroke in T2DM mice compared to in their lean controls. Importantly, all abnormal changes were significantly prevented by rFGF21 administration initiated at 6 h after stroke. Our in vitro experimental results also demonstrated that rFGF21 protects against hyperglycemia plus interleukin (IL)-1β-induced transendothelial permeability through upregulation of junction protein expression in an FGFR1 activation and PPARγ activity elevation-dependent manner. Our data suggested that rFGF21 has strong protective effects on acute BBB leakage after diabetic stroke, which is partially mediated by increasing PPARγ DNA-binding activity and mRNA expression of BBB junctional complex proteins. Together with our previous investigations, rFGF21 might be a promising candidate for treating diabetic stroke.

## 1. Introduction

About 30% of stroke patients are diabetic, and more than 90% of them have type 2 diabetes mellitus (T2DM) [1]. T2DM stroke patients have nearly double the mortality of nondiabetic stroke patients and worse neurological outcomes [2,3]. Better understanding of specific pathophysiology and development of targeted therapies for T2DM stroke patient subgroups have become a high priority in translational stroke research [4].

Despite a large body of data demonstrating T2DM-induced microvascular complications in the kidney and retina, the impact of T2DM on the cerebrovasculature is still poorly understood [5]. Emerging evidence obtained from clinical and experimental studies suggests that T2DM impairs blood–brain barrier (BBB) integrity and aggravates BBB permeability in baseline state or after ischemic stroke [5,6,7,8], which has been considered an important pathological feature of T2DM stroke responsible, at least in part, for the worsening of neurological deficits [9]. We have previously proposed a disease-modifying approach using recombinant fibroblast growth factor 21 (rFGF21) to treat ischemic stroke mice with T2DM and observed the beneficial effects of rFGF21 on long-term neurological outcomes in T2DM stroke mice [10]. Very interestingly, FGF21 has been reported as a key mediator involving in the physiological and pharmacological actions of peroxisome proliferator-activated receptor gamma (PPARγ) [11], which is an important nucleus receptor that transcriptionally regulates multiple gene expressions and functions as a master gatekeeper for cytoprotective stress response, such as anti-inflammation and antioxidative stress, in poststroke brain injury and repair [12]. Our previous study showed that rFGF21 administration increases PPAR*γ* DNA-binding activity three days after stroke in the perilesion cortex of T2DM mice, which might be partially responsible for the reduction of brain tissue damage and detrimental proinflammatory cytokine expressions [10]. Others have reported that PPARγ activity in brain tissue is dramatically declined after ischemic stroke, which leads to downregulation of tight junction (TJ) proteins and subsequent BBB leakage [13,14]. However, pharmacological effects of rFGF21 on aggravated early BBB disruption after ischemic stroke with T2DM and its potential underlying molecular mechanisms have not been investigated.

In this study, we tested our hypothesis that poststroke administration of rFGF21 is protective against early BBB damage in T2DM mice via FGFR1-mediated elevation of cerebrovascular PPAR*γ* activity. Two sets of experiments were designed as followed: in vivo study was performed using a focal stroke model in T2DM mice, treated with or without rFGF21 as we previously described [10], and an in vitro study was conducted using cultured human brain microvascular endothelial cells (HBMECs), insulted by a well-established hyperglycemia plus interleukin (IL)-1β exposure model to mimic in vivo situation of diabetic stroke as we previously described [15].

## 2. Results

### 2.1. rFGF21 Increases PPARγ DNA-Binding Activity via FGFR1 at a Peri-infarct Area after Distal Middle Cerebral Occlusion (dMCAO) in db/db Mice

**Figure 1:**
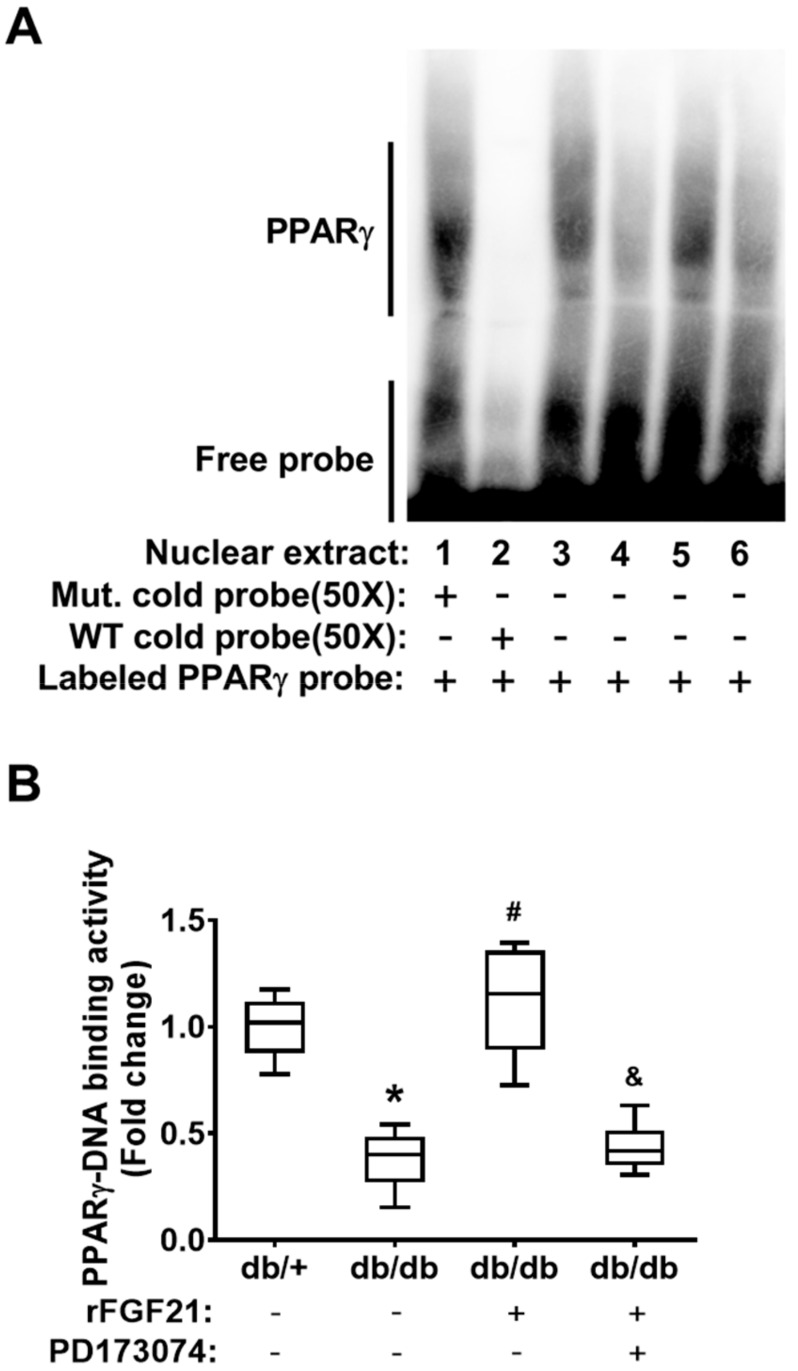
Recombinant fibroblast growth factor 21 (rFGF21) increases peroxisome proliferator-activated receptor gamma (PPARγ) DNA-binding activity via FGFR1 at a peri-infarct area after distal middle cerebral occlusion (dMCAO) in db/db mice. At 24 h poststroke, the transcriptional factor PPARγ DNA-binding activity in nuclear fractions was measured by an electrophoresis mobility shift assay (EMSA). (**A**) Representative image of the EMSA gel. The order of sample loading: lanes 1–3 were for all db/+ stroke, lane 4 was for db/db stroke, lane 5 was for db/db stroke + rFGF21, and lane 6 was for db/db stroke + rFGF21 + PD173074. (**B**) Densitometric quantification of specific PPARγ DNA-binding bands. Data are illustrated as box-plots with the median, lower and upper quartiles, minimal and maximal value (*n* = 6 per group). * *p* < 0.05 for db/db stroke vs. db/+ stroke; # *p* < 0.05 for db/db stroke + rFGF21 vs. db/db stroke; ^&^ *p* < 0.05 for db/db stroke + rFGF21 + PD173074 vs. db/db stroke + rFGF21.

First, we examined the alteration of brain tissue PPARγ activity in post-dMCAO db/db mice. Due to a very limited amount of nuclear fraction extracted from mouse brain microvascular fragments, we have difficulty directly assessing the cerebrovascular PPARγ activity. Instead, we analyzed PPARγ DNA-binding activity in the nuclear fraction from peri-infarction brain tissue at 24 h post-dMCAO using an electrophoresis mobility shift assay (EMSA) (Figure 1A). Our results showed that PPARγ DNA-binding activity was markedly reduced (62.2% reduction) in db/db mice compared to that in db/+ mice, demonstrating the impaired poststroke PPARγ activity in the context of diabetic stroke (Figure 1B). Importantly, the delayed rFGF21 administration significantly rescued the decline in poststroke PPARγ DNA-binding activity (196.1% increase compared to in the nontreated group) in db/db mice. However, the treatment at 30 min before rFGF21 administration with PD173074 significantly abolished the effect of rFGF21 on promoting PPARγ DNA-binding activity (Figure 1B), suggesting the PPARγ activation induced by rFGF21 treatment is mediated by FGFR1.

### 2.2. rFGF21 Reduces BBB Extravasation via PPARγ Activation at a Peri-Infarct Area after dMCAO in db/db Mice

**Figure 2:**
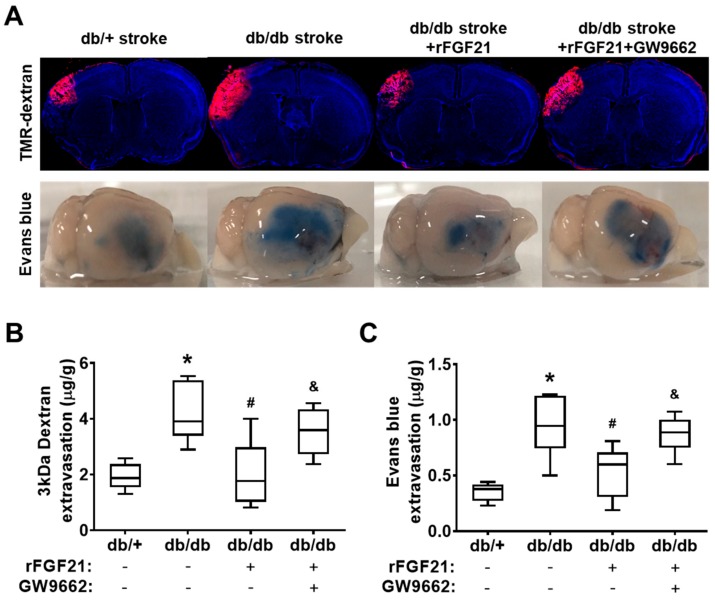
rFGF21 reduces blood–brain barrier (BBB) extravasation via PPARγ activation at a peri-infarct area after dMCAO in db/db mice. At 48 h poststroke, extravasations of 3 kDa TMR-dextran and Evans blue dye were measured by a spectrometer. (**A**) Representative images of 3 kDa TMR-dextran and Evans blue extravasations. (**B**) Quantification of 3 kDa TMR-dextran extravasation. (**C**) Quantification of Evans blue dye extravasation. Data are illustrated as box-plots with the median, lower and upper quartiles, minimal and maximal value (*n* = 6 per group). * *p* < 0.05 for db/db stroke vs. db/+ stroke; # *p* < 0.05 for db/db stroke + rFGF21 vs. db/db stroke; ^&^ *p* < 0.05 for db/db stroke + rFGF21 + GW9662 vs. db/db stroke + rFGF21.

Effects of rFGF21 on poststroke BBB leakage were tested by a BBB extravasation assay using two different tracers: TMR-dextran (3 kDa) and Evans blue dye (approximately 68 kDa after binding with albumin in the circulation) (Figure 2A). At 48 h after dMCAO, as we expected, the extravasation assay showed a dramatic increase in BBB leakage in db/db mice compared to in db/+ mice as controls (117.6% increase of 3 kDa TMR-dextran and 167% increase of Evans blue dye) (Figure 2B,C). Importantly, rFGF21 administered at 6 h after stroke onset significantly lowered the leakage of both tracers in db/db mice (52% reduction in 3kDa TMR-dextran leakage and 43.2% reduction in Evans blue dye leakage). However, the treatment at 30 min prior to rFGF21 administration with GW9662 eliminated the inhibitory effect of rFGF21 on BBB extravasation (Figure 2B,C). These results suggested that the potentiated BBB leakage after ischemic focal stroke in T2DM can be largely (about 50%) protected by rFGF21 and this beneficial effect might be contributed by PPARγ activation.

### 2.3. rFGF21 Inhibits Reduction of Junction Protein mRNA Expression via Cerebrovascular PPARγ Activation after dMCAO in db/db Mice

**Figure 3:**
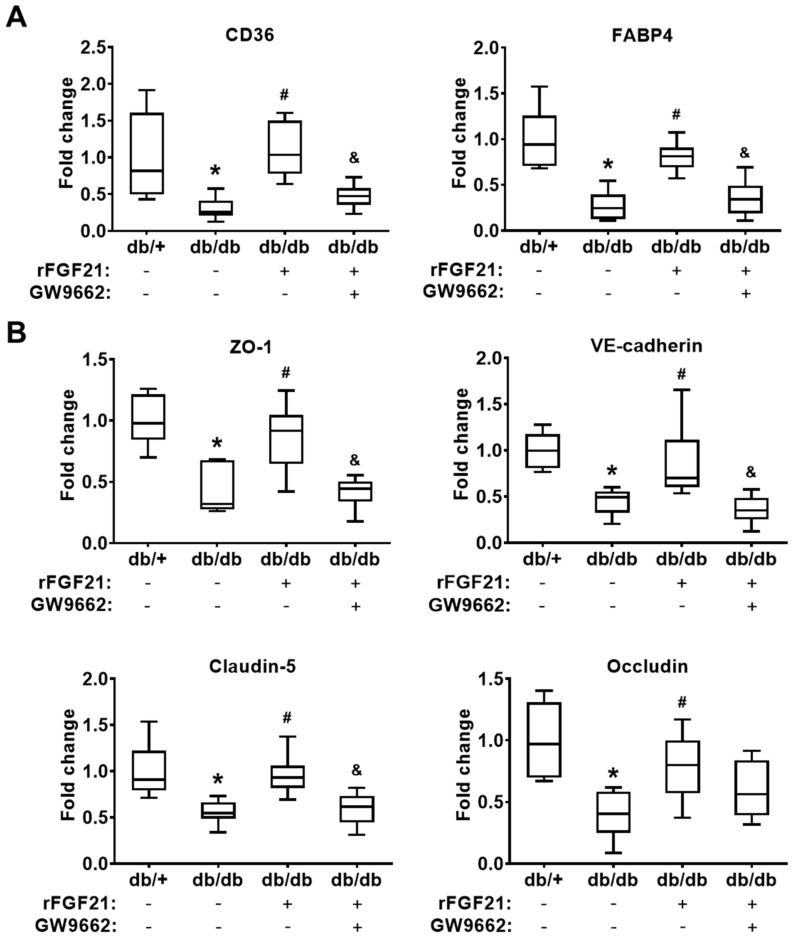
rFGF21 inhibits reduction of junction protein mRNA expression via cerebrovascular PPARγ activation after dMCAO in db/db mice. At 24 h poststroke, mRNA levels of CD36, FABP4, ZO-1, VE-cadherin, claudin-5, and occludin were measured by RT-PCR. (**A**) Relative fold changes of PPARγ-targeted genes *CD36* and *FABP4*. (**B**) Relative fold changes of junction protein genes *ZO-1*, VE-cadherin, claudin-5, and occludin. Data are illustrated as box-plots with the median, lower and upper quartiles, minimal and maximal value (*n* = 6 per group). *p < 0.05 for db/db stroke vs. db/+ stroke; # *p* < 0.05 for db/db stroke + rFGF21 vs. db/db stroke; ^&^ *p* < 0.05 for db/db stroke + rFGF21 + GW9662 vs. db/db stroke + rFGF21.

In the last part of the in vivo experiments, we indirectly evaluated the cerebrovascular PPARγ activity by quantitating mRNA expression levels of two PPARγ-targeted downstream genes *CD36* and *FABP4* [16,17] in cerebral microvascular fragments 24 h after ischemic focal stroke. Our results showed there were significant reductions of both PPARγ downstream genes after stroke (there was 96.8% reduction in *CD36* mRNA level and 73% reduction in *FABP4* mRNA level) in db/db mice compared to those in db/+ mice. The rFGF21 administration given 6 h after stroke eliminated this reduction (263.8% increase in *CD36* and 199.6% increase in *FABP4*) (Figure 3A), which reflected the change of cerebrovascular PPARγ activity. To investigate the effects of rFGF21 on BBB junction protein expression, the same mRNA samples extracted from isolated cerebral microvascular fragments were used for RT-PCR assays. There were dramatic decreases in mRNA levels of BBB junction proteins, including ZO-1 (57.7% reduction), VE-cadherin (55.1% reduction), claudin-5 (44.4% reduction), and occludin (60.2% reduction) in db/db mice compared to in db/+ mice. Very importantly, a delayed rFGF21 administration largely ameliorated the reduction of the junction protein mRNA expression, but this protective effect was blocked by a specific PPARγ inhibitor GW9662 (Figure 3B). These results suggested that protection provided by rFGF21 against poststroke BBB disruption might be, at least in part, mediated by promoting cerebral microvascular PPARγ activity and PPARγ activity-associated BBB junction protein expression.

### 2.4. rFGF21 Ameliorates Transendothelial Permeability via Promoting FGFR1-mediated PPARγ Activity in Cultured HBMECs

**Figure 4:**
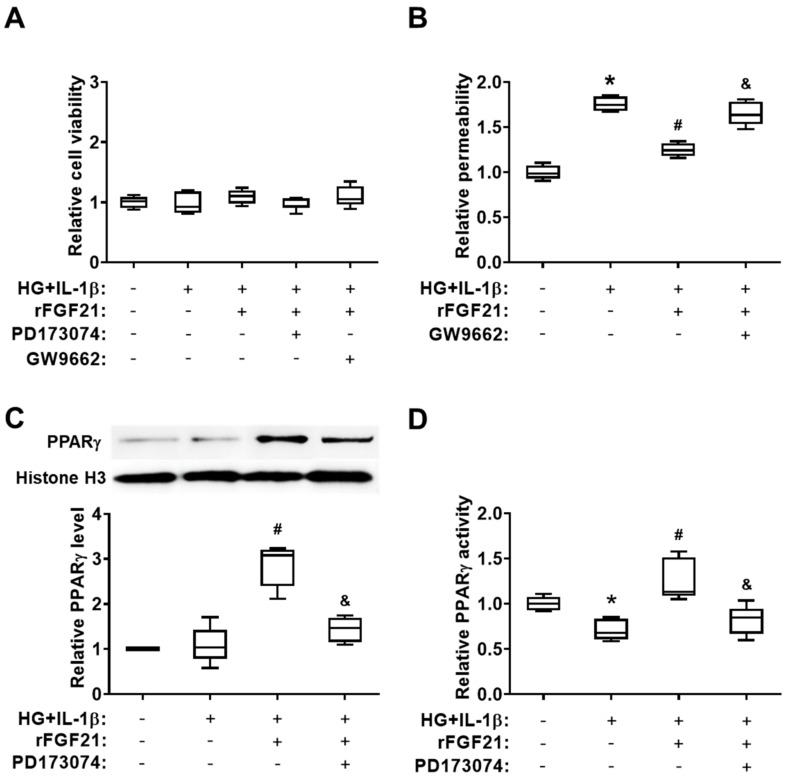
rFGF21 ameliorates transendothelial permeability via promoting FGFR1-mediated PPARγ activity in cultured human brain microvascular endothelial cells (HBMECs). HBMECs were cultured under hyperglycemia condition for 3 days and then exposed to interleukin (IL)-1β for 16 h (HG + IL-1β), either in regular plates for cell viability assays or in Transwells plates for monolayer permeability assessments. Cell viability was measured by WST-1 assay, and cell permeability of fluorescein isothiocyanate (FITC)-labeled dextran was assessed by a fluorescence plate reader. In two separate experiments, protein samples were collected for Western blots and PPARγ activity assay. (**A**) Cell viability of cultured HBMECs after different treatments. (**B**) Relative fold change of FITC-dextran permeability in cultured HBMECs. (**C**) Representative Western blot image and quantification of protein level of PPARγ in cultured HBMECs. (**D**) Relative fold change of PPARγ activity. Data are illustrated as box-plots with the median, lower and upper quartiles, minimal and maximal value (*n* = 5 per group). * *p* < 0.05 for injured vs. normal; # *p* < 0.05 for injured + rFGF21 vs. injured; ^&^ *p* < 0.05 for injured + rFGF21 + GW9662 or PD173074 vs. injured + rFGF21.

In the second set of experiments, we used cultured HBMECs to further test our hypothesis. Hyperglycemia (HG) plus IL-1β exposure was applied to mimic diabetic stroke conditions in vitro. We also measured the permeability of a cultured HBMEC monolayer after HG + IL-1β insult. Firstly, we found there was no significant change of cell viability between each group of cultured HBMECs by applying different conditions (Figure 4A). Subsequently, cultured HBMEC monolayer permeability after the injury by hyperglycemia plus IL-1β was measured (Figure 4B), and, as we expected, cell permeability was dramatically increased after injury (75.7% elevation compared to that in the noninjured group) while the rFGF21 treatment significantly reduced cell permeability by 28.9% compared to that in the nontreated group. Again, the GW9662 co-treatment eliminated the protective effect of rFGF21 (32.4% elevation). Furthermore, the HG + IL-1β insult induced a decrease in PPARγ activity (28.8% reduction compared to that in the noninjured group) in nuclear fraction of cultured HBMECs, and rFGF21 increased not only the protein level (162.5%) but also the activity (77.9%) of PPARγ in cultured HBMECs after the injury compared to those in the nontreated group. While the co-treatment with an FGFR1 inhibitor PD173074 significantly impaired the action of rFGF21 in upregulating the protein level (50% reduction) and activity (35.7% reduction) of PPARγ in injured HBMECs, suggesting the involvement of FGFR1 in the PPARγ-regulatory function of rFGF21 (Figure 4C,D). Consistently, our previous data also showed that phosphorylation and activation of FGFR1 might be an essential step in exerting the protective functions of rFGF21 in HBMECs [15].

### 2.5. rFGF21 Increases Junction Protein Expression via Promoting PPARγ in Cultured HBMECs

**Figure 5:**
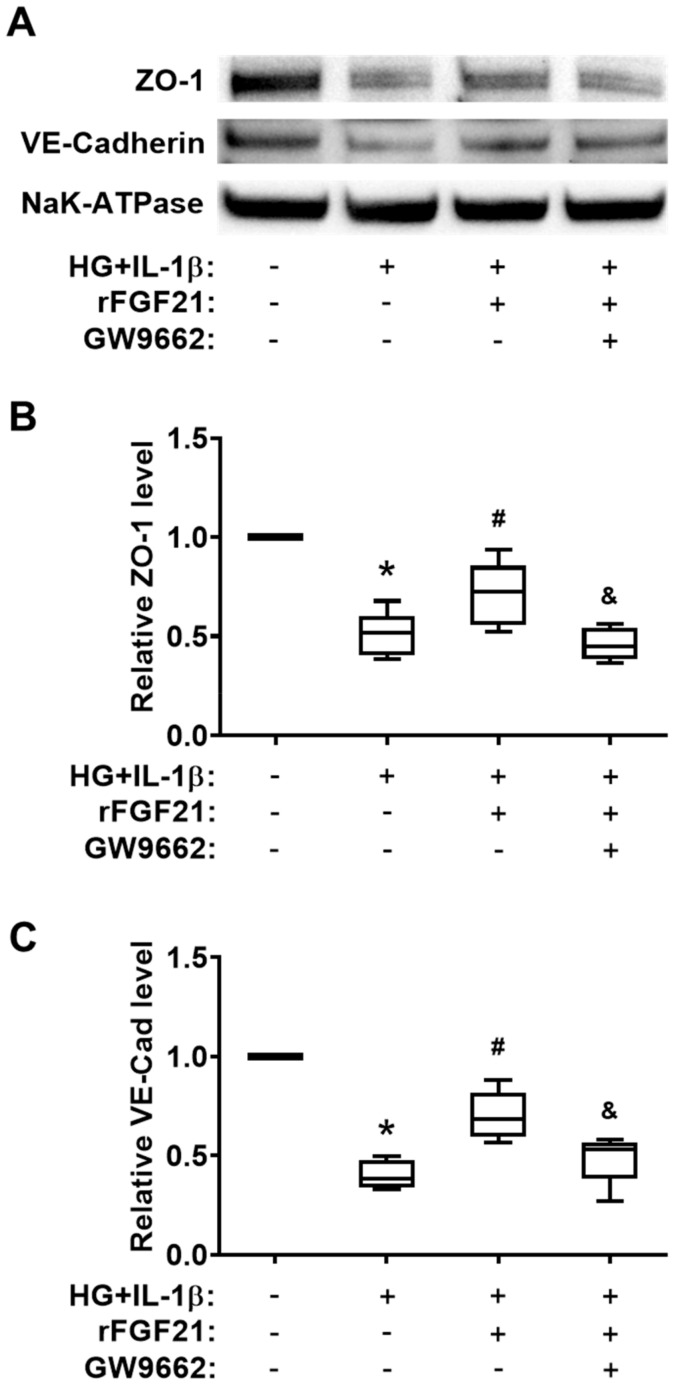
rFGF21 increases junction protein expression via promoting PPARγ in cultured HBMECs. HBMECs were cultured under hyperglycemia for 3 days and then exposed to IL-1β for 16 h (HG + IL-1β), and membrane proteins fraction was isolated for Western blots. (**A**) Representative Western blot gel images of ZO-1 and VE-cadherin expression. (**B**) Relative fold change of junction protein ZO-1 in cultured HBMECs. (**C**) Relative fold change of junction protein VE-cadherin in cultured HBMECs. Data are illustrated as box-plots with the median, lower and upper quartiles, minimal and maximal value (*n* = 5 per group). * *p* < 0.05 for injured vs. normal; # *p* < 0.05 for injured + rFGF21 vs. injured; ^&^ *p* < 0.05 for injured + rFGF21 + GW9662 vs. injured + rFGF21.

We quantified the protein levels of two junction proteins, ZO-1 and VE-cadherin, in cultured HBMECs under HG + IL-1β conditions (Figure 5A). Both protein levels were significantly decreased after HG + IL-1β injury (49.4% reduction of ZO-1 and 59.6% reduction of VE-cadherin compared to those in the noninjured group), while the rFGF21 treatment significantly upregulated the protein levels of ZO-1 (40.3% increase) and VE-cadherin (73.9% increase) in injured HBMECs (Figure 5B,C). Again, the GW9662 co-treatment impeded the elevation of junction proteins by rFGF21 (35.2% and 30.7% reductions, respectively), suggesting the participation of PPARγ in regulation of TJ protein expression by rFGF21 in cultured HBMECs.

## 3. Discussion

**Figure 6:**
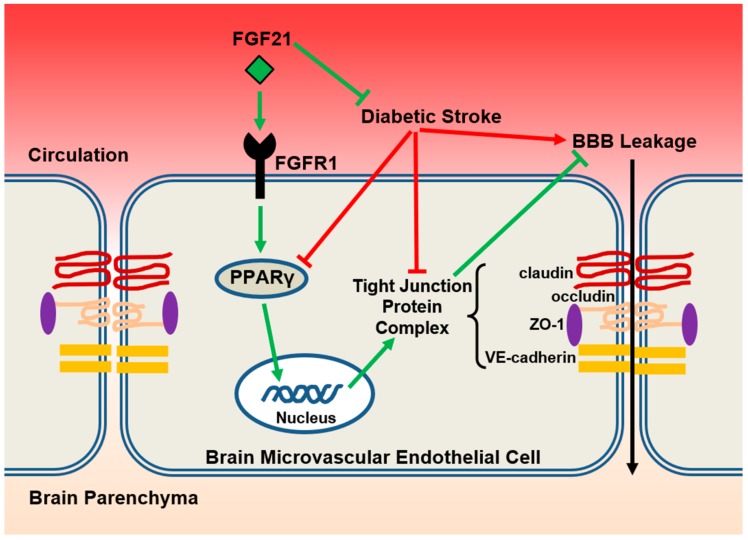
The proposed model. In the setting of diabetic ischemic stroke, brain microvascular endothelial cells suffer more severe damage, including exacerbated reduction of PPARγ activity and tight junction protein expressions, and subsequently augmented BBB leakage. Exogenous administration of rFGF21 exerts BBB-protective effects through FGFR1-mediated elevation of PPARγ activity and upregulation of tight junction protein expressions and eventually mitigates the poststroke BBB permeability in the context of type 2 diabetes mellitus (T2DM).

The major findings of the present study can be summarized as follows: (1) T2DM db/db mice exhibited much worse BBB permeability in acute phase after focal ischemic stroke compared to their lean db/+ counterparts; (2) delayed rFGF21 administration given 6 h after stroke onset largely mitigated the ischemia-induced BBB permeability in db/db mice; (3) the BBB-protective effect of rFGF21 was at least partially contributed by FGFR1-mediated promotion of PPARγ activity in cerebral microvascular endothelium (Figure 6).

Fibroblast growth factor 21 (FGF21) is an endocrine member of the FGF family, playing a potent and central role in glucose and lipid metabolism as well as in energy balance [18]. FGF21 also mediates adaptive responses to tissue damage and repair in various pathological conditions [19,20,21]. Using the same type 2 diabetes animal model, db/db mice and nondiabetic genetic control db/+ mice, we previously tested our hypothesis that rFGF21 might be a novel and potent disease-modifying approach to improving neurological outcomes after T2DM stroke. Our earlier explorative investigation suggests that the potential therapeutic mechanisms of rFGF21 are, at least in part, achieved by systemic metabolic modulation, inhibition of pro-neuroinflammation, and promotion of whiter matter integrity [10]. However, protective effects of rFGF21 on acute brain injury, especially aggravated BBB integrity disruption in T2DM stroke, which might play a critical role in its therapeutic benefits, have not been investigated.

It has been well-known that postischemic BBB damage leads to a dynamic, biphasic change of its permeability. Previous experimental studies using rodent stroke models have demonstrated that two peaks of BBB leakage, an early extravasation (3–6 h after stroke) followed by a late one (24–48 h after stroke) [22,23,24,25], exist after stroke. Since we initiated rFGF21 treatment 6 h after stroke, BBB extravasation based on using two tracers with different molecular weights (3 kDa TMR-dextran and Evans blue dye, which is approximately 68 kDa after binding with albumin) was examined 48 h after stroke in this study.

In the first set of experiment, we found markedly increased leakages of both tracers through BBB in db/db mice after stroke compared to in db/+ mice. Importantly, the delayed poststroke rFGF21 administration significantly lowered (about 50% reduction) the BBB leakage in db/db mice. Next, we selected PPARγ as our major target to investigate the therapeutic mechanisms underlying rFGF21 treatment in T2DM stroke, since PPARγ has been well-known for its pivotal role in anti-inflammation, antioxidative stress, and subsequent vascular protection [26]. Due to very limited amount of nuclear fraction extracted from brain microvascular fragments, we have difficulty directly examining the PPARγ activity in cerebrovascular nuclear fraction. Instead, we used EMSA to test PPARγ DNA-binding activity in the nuclear fraction obtained from brain tissue at 24 h post-dMCAO. We found a severe decline in PPARγ activity after stroke in db/db mouse brain compared to in db/+ mice, but it was almost completely rescued by the delayed rFGF21 administration. The RT-PCR analysis showed poststroke declines in the mRNA expression of PPARγ-targeted downstream genes *CD36* and *FABP4* and endothelial junction proteins in cerebral microvascular fragments of db/db mice were largely prevented by rFGF21 administration, suggesting that rFGF21 targets specific pathological responses in cerebral microvasculature after T2DM stroke. To explore molecular mechanisms, which is based on our previous data showing rFGF21 may signal through FGFR1 phosphorylation and activation to exert its functions [15,27], we tested the possible involvement of FGFR1 in the change of PPARγ activity by pharmacological approaches including a specific FGFR1 inhibitor PD173074 and a specific PPARγ inhibitor GW9662 separately. Our in vivo results indicated a possible cascade, i.e., FGF21–FGFR1–PPARγ activation, underlying the pharmacological action of FGF21 may be involved in the regulation of junction protein expression and subsequent BBB protection in T2DM db/db mice after stroke. In the second set of experiments, we used cultured HBMECs to further test our hypothesis. Hyperglycemia plus IL-1β simulated diabetic stroke insult in vitro as we previously described [15]. Our in vitro experimental results demonstrated that rFGF21 is strongly protective in hyperglycemia plus IL-1β -induced transendothelial permeability, which is FGFR1 activation-dependent and PPARγ activity-dependent, and also associated with the maintenance of junction protein expression. These results clearly defined the pharmacological action cascade, that is, FGF21–FGFR1–PPARγ activation–BBB junction protein expression–endothelial leakage protection.

It has been reported that the very low heparin-binding affinity makes FGF21 capable of crossing BBB by simple diffusion [28], which has been also confirmed by our previous experiment in high-fat diet-fed mice [27]. However, as a large molecule protein, cerebrovascular effects of FGF21 might act dominantly in the context of neurovascular unit. Enormous clinical and experimental investigations have demonstrated that BBB integrity disruption is a key cerebral vascular pathology primarily mediating acute brain injury after ischemic stroke [29,30] and associated with poor prognosis poststroke [31]. Knowledge regarding diabetic-associated brain microvasculopathy is still incomplete, and several mechanisms may synergistically lead to the BBB dysfunction in diabetic state, such as long-term hyperglycemia-induced endothelial anatomical and metabolic changes, oxidative stress, and deranged inflammatory responses [5,32]. Most importantly, emerging experimental investigations have shown a significantly exacerbated poststroke BBB disruption in T2DM, which has been considered one of pathological features of T2DM stroke and therapeutic targets [33,34].

In this study, we found a significant decrease in PPARγ DNA-binding activity in a perilesion cortex, as well as mRNA expression of PPARγ-targeted downstream genes in cerebral microvascular fragment at one day after stroke in T2DM mice, but it was significantly elevated by the poststroke administration of rFGF21. Although, as a transcriptional factor, how the rFGF21-activated PPARγ regulates subesequent signaling pathways in modulating junction protein expression and preserving BBB integrity remains to be elucidated, accumulating experimental evidence has supported the critical roles of PPARγ activity in not only BBB protection, but also improvement of cellular survival and recovery of homeostatic equilibrium after ischemic stroke [12]. It is possible that other molecular mechanisms may be also involved in poststroke BBB protection by rFGF21, which needs to be investigated in the future. The present study confirmed the greatly aggravated BBB damage after focal ischemic stroke in T2DM mice and, most importantly, the potent early BBB-protective effect of delayed rFGF21 administration, which might be one of key beneficial actions for improving long-term neurological outcome, and together with a delayed therapeutic time window, indicating that rFGF21 might be a highly translational candidate for treating stroke with T2DM. Indeed, the number of diabetic patients is expected to sharply increase within the next decade. By 2030, the estimated prevalence of diabetes mellitus worldwide will exceed 437 million [35], and the incidence rate of diabetes-related vascular complications will also rise dramatically, including acute ischemic stroke [36]. Particularly, these patients may suffer from more severe ischemic brain damage and worse outcome [37,38] due to long-term systemic vascular abnormalities, including the changes of BBB [6]. Our study may provide a new thought for ameliorating BBB damage in diabetic stroke via a disease-modifying approach and eventually benefit this subgroup of patients.

We are aware of some limitations in this study. First, we used C57BLKS-Leprdb type 2 diabetes mice (db/db T2DM mice, Jackson Lab); however, the leptin receptor mutation does not accurately reflect the true pathogenesis of the disease in humans, even if this model has already given us an insight into glucose metabolism and novel pathways of vascular complications under diabetic state [39,40]. Further tests using rFGF21 in other animal models of diabetes with ischemic stroke should be pursued [41]. Second, we tested effects of rFGF21 on PPARγ activation-associated BBB protection after focal stroke in T2DM mice. Nevertheless, detailed molecular mechanisms for FGF21-induced PPARγ activation and downstream signaling pathways of PPARγ activation in modulating BBB integrity after T2DM stroke need to be elucidated in future investigations. Third, there are multiple pathological factors that dynamically and interactively participate in T2DM stroke-induced impairment of BBB integrity [42]. Thus, it would be very important to understand how rFGF21 may pharmacologically modulate these pathological mechanisms, which requires more investigations [43]. Fourth, due to lack of available working antibodies for immunohistochemistry to detect the active form of the FGF21 receptor FGFR1 (phosphorylated FGFR1), we were unable to define the specific regions inside the mouse brain, where the FGF21-FGFR1 activation took place. Indeed, the FGF21-mediated receptor activation and consequent biological signaling pathways in the context of neurovascular unit warrant further investigation [44]. Fifth, although permeability assays based on in vitro cultured endothelial monolayers have been commonly used, it may not comprehensively reflect the physiological features of cerebrovascular endothelium in vivo. Thus, endothelium–astrocytes co-culture may be a better in vitro model for our future studies [45]. Lastly, the present study is our continuing investigation for rFGF21 therapeutic effects in T2DM mice with focal ischemic stroke. As we previously demonstrated that multiple pharmacological actions of rFGF21 may involve in this therapeutic process, it is impossible to truly determine the portion, of which the improved neurological outcomes are exclusively mediated by BBB-protective effects of rFGF21. The much worse BBB damage and potent protective effects of rFGF21 suggest the BBB protection mechanism may at least in part contribute to its long-term neurological outcome improvement after ischemic focal stroke in T2DM mice [10].

## 4. Materials and Methods

### 4.1. Mouse Model of Focal Cerebral Ischemia

Male BKS.Cg-Dock7m +/+ Lepr db/J homozygous (db/db) mice at 11–12 weeks of age, weighing 40–50 g, and their age-matched nondiabetic counterparts BKS.Cg-Dock7m +/+ Lepr db/J heterozygous (db/+) mice, weighing 25–30 g, were obtained from the Jackson Laboratory. Mice were housed in a standard animal facility with a 12 h dark-light cycle and fed with pellets and water ad libitum. Focal cerebral ischemia was established by the coagulation of the distal middle cerebral artery (dMCAO), followed by 90 min common ipsilateral carotid artery occlusion as we previously described [10]. All experiments were performed after protocols approved by the Massachusetts General Hospital (2016N000498, 02 08 2019) and Tulane University (846, 21 11 2019) Institutional Animal Care and Use Committee in compliance with the NIH Guide for the Care and Use of Laboratory Animals.

### 4.2. Cultured HBMECs and Injury Model

Primary HBMECs were purchased from Cell Systems Corporation (ACBRI376, Kirkland, WA, USA) and cultured in complete growth media EBM-2 containing supplemental growth factors (Lonza). To mimic the in vivo environment of diabetic stroke, we applied a cell injury model using hyperglycemia plus IL-1β in cultured HBMECs as we previously published [15]. Briefly, primary HBMECs (culture or monolayer) was initially cultured in normal complete EBM-2 (with 5 mM glucose) and then transferred into hyperglycemic (25 mM glucose) EBM plus IL-1β (20 ng/mL) for 16 h. An HBMEC monolayer was established by culturing primary HBMECs on the inner surface of collagen-coated Transwell inserts (with a 6.5 mm diameter and a 0.4 μm pore size; Corning, New York, NY, USA). The viability of cultured HBMECs was assessed by WST-1 assay [15]. Cells were incubated at 37 °C for 2 h after adding a cell proliferation reagent WST-1 (Donjindo Molecular Technologies, Rockville, MD, USA) into 24-well plates containing a 450 μL medium in each well. The absorbency at 450 nm was measured by using a microplate reader (SpectraMax M5, Molecular Devices, San Jose, CA, USA) against a blank background control.

### 4.3. Experimental Groups

After receiving dMCAO, mice were randomly assigned to four experimental groups: (1) nondiabetic db/+ mice (db/+); (2) diabetic db/db mice (db/db); (3) db/db mice receiving rFGF21; and (4) db/db mice receiving rFGF21 plus GW9662 or PD173074. rFGF21 was dissolved in saline and intraperitoneally administered at 6 h after stroke twice per day until sacrifice (1.5 mg/kg per single injection) [10]. PD173074 (15 mg/kg, Sigma, Burlington, MA, USA), a specific FGFR1 inhibitor, or GW9662 (4 mg/kg, Sigma), a selective antagonist for a transcriptional factor PPARγ, was dissolved in 40% (2-hydroxypropyl)-β-cyclodextrin and intraperitoneally administered at 30 min prior to the rFGF21 treatment. Groups 1 and 2 were also intraperitoneally administered the same amount of 40% (2-hydroxypropyl)-β-cyclodextrin. For in vitro studies, five groups were included: (1) sham group without any treatment; (2) hyperglycemia and IL-1β group; (3) HG + IL-1β group plus rFGF21 (added into a medium at a final concentration of 50 nM during IL-1β treatment; (4) HG + IL-1β + rFGF21 group co-treated with PD173074 (50 nM) or GW9662 (20 nM) dissolved in DMSO. Dose selection of GW9662 and PD173074 was based on previous published studies [15,46,47,48].

### 4.4. Mouse BBB Permeability Assay

Mouse BBB permeability assay was performed as previously described [49,50] with minor modifications. Briefly, at 48 h poststroke, mice were intravenously injected with 3 kDa TMR-dextran (1 μg/μL with a total volume of 200 μL dissolved in saline, Thermo Fisher) or Evans blue dye (2% with a total volume of 200 μL dissolved in saline, Sigma). After circulation for 1 h (3 kDa TMR-dextran) or 30 min (Evans blue dye) separately mice were deeply anesthetized and transcardially perfused with adequate ice-cold PBS immediately following the blood collection from the left ventricle. Peri-infarct brain tissue was harvested and homogenized in ice-cold PBS with 1% Triton X-100 (for 3 kDa TMR-dextran) or 50% trichloroacetic acid (for Evans blue dye), followed by centrifugation. The fluorescence in supernatants was read by a microplate reader at excitation and emission wavelengths of 540 and 590 nm, respectively (for 3 kDa TMR-dextran) or of 630 and 680 nm, respectively (for Evans blue dye). Values of tracer content in the brain tissue (μg/g) were corrected by fluorescence readings from the standard and the serum.

### 4.5. Endothelial Monolayer Permeability Assay

After treating each group of HBMEC monolayers with different conditions, the media in both upper and lower chambers were then replaced by fresh media without supplement. Permeability was assessed by adding fluorescein isothiocyanate (FITC)-labeled dextran (0.1 mg/mL, molecular weight: 70 kD, Sigma) to the upper chamber, while the lower compartment containing a 500 μL fresh serum-free medium was incubated for 20 min. We then collected a 100 μL medium from the lower chamber and measured the fluorescence emitted at 520 nm with an excitation wavelength of 490 nm.

### 4.6. Nuclear Extraction and EMSA

We collected peri-infarct brain tissue at 24 h poststroke for nuclear fraction extraction by using A commercially available kit (Sigma) and subsequently used for EMSA as we previously described [10]. Briefly, the oligonucleotides bearing wild-type or mutated putative transcription-factor-binding sites were synthesized as follows: PPARγ consensus oligonucleotide (sense): 5’-CAAAACTAGGTCAAAGGTCA–3’, PPARγ consensus oligonucleotide (antisense): 5’-TGACCTTTGACCTAGTTTTG–3’, PPARγ mutant oligonucleotide (sense): 5’-CAAAACTAGCACAAAGCACA–3’, and PPARγ mutant oligonucleotide (antisense): 5’-TGTGCTTTGTGCTAGTTTTG–3’ (Massachusetts general hospital DNA core facility). Double-stranded DNA probes were obtained from annealing of synthetized single-stranded oligonucleotides labeled with biotin at 5′-end. We used the LightShift EMSA Optimization and Control Kit (Pierce, Rockford, IL, USA) to perform DNA–protein binding reactions, followed by detection of biotin-labelled DNA using Chemiluminescent Nucleic Acid Detection Module (Pierce). Quantitative densitometry was performed on identical bands by using ImageJ software (1.52i, NIH, Bethesda, MD, USA).

### 4.7. Mouse Brain Microvascular Endothelial Cell Isolation

At 24 h poststroke, mice were sacrificed for brain microvascular endothelial isolation as described by others [51] with minor modifications. Briefly, the entire procedure was performed on ice, and the brain tissue was homogenized and then centrifuged at 2000× *g* for 15 min at 4 °C to carefully separate the myelin and subsequently collect the vessel pellet. Samples were stored at −80 °C before the use.

### 4.8. RT-PCR Assay

Mouse brain microvascular endothelial cell isolation was used for obtaining total RNA using the miRNeasy Micro Kit (Qiagen), and RT-PCR) was performed as we previously described [10]. We used Taqman gene expression assays for *CD36* (Mm00432403_m1), *FABP4* (Mm00445878_m1), *ZO-1* (Mm00493699_m1), *VE-cadherin* (Mm00486938_m1), claudin-5 (Mm00727012_s1), occludin (Mm00500912_m1), and the house keeping gene *B2M* (Mm00437762_m1) (Appiled Biosystems). Data were normalized by the quantification of the endogenous 18S ribosomal RNA. Relative mRNA expression levels were calculated by the 2^−ΔΔCt^ method and presented by fold change.

### 4.9. PPARγ Activity in Vitro Assay

PPARγ activity was measured using PPAR gamma Transcription Factor Assay Kit (ab133101, Abcam) following the manufacturer’s instructions. Briefly, nuclear extracts were isolated from cultured HBMECs using “Nuclear Extraction Kit” (ab113474, Abcam). Ten microliters of nuclear extract from HBMECs or positive control was mixed with 90 µL Complete Transcription Factor Binding Assay Buffer (CTFB) and added to designated wells of a 96-well assay plate. The plate was then sealed and incubated at 4 °C overnight. The next day, the wells were emptied and washed five times with 200 µL of Wash Buffer. Transcription Factor PPAR gamma Primary Antibody (100 µL, 1:100) was then added to each well and incubated at room temperature for 1 h without agitation. After five times wash, goat anti-Rabbit HRP conjugate (1:100) was added to each well, followed by 1 h incubation at room temperature. After washing, a 100 µL developing solution was added and incubated at room temperature for 15 to 45 min, followed by adding a 100 µL stop solution. Absorbance at 450 nm was measured using a plate reader.

### 4.10. Western Blot

For total protein isolation of cultured HBMECs, cells were washed twice with PBS prior to adding a lysis buffer. A plasma membrane protein extraction kit (Abcam, Cambridge, MA, USA) was used for sample collection. Protein samples (plasma membrane proteins or nuclear extractions) were equally separated in a 4%–20% NuPage gel prior to being transferred to nitrocellulose membranes. Primary antibodies used for overnight membrane incubation at 4 °C are as follows: PPARγ (1:1000, Thermo Fisher, Rockford, IL, USA), ZO-1 (1:1000, Cell Signaling, Beverly, MA, USA), VE-cadherin (1:1000, Enzo Life Sciences, Farmingdale, NY, USA), Na-K-ATPase (1:1000, Abcam), and Histone H3 (1:1000, Cell Signaling). Membranes were then incubated with horseradish peroxidase-linked secondary antibodies (1:2000) for 1 h at room temperature after being washed in PBS containing 0.1% Tween 20 and developed by enhanced chemiluminescence (Pierce). The optical density of protein bands was quantified with ImageJ.

### 4.11. Statistical Analysis

All data were illustrated as box-plots with the median, lower and upper quartiles, minimal and maximal values, and analyzed in GraphPad Prism version 7.0 using one-way ANOVA plus Tukey–Kramer’s test between groups. Statistical significance was defined as *p* < 0.05.

## 5. Conclusions

In summary, we demonstrated that rFGF21 administration is potently beneficial for protection against early BBB integrity disruption in T2DM stroke mice. The potential mechanisms are, at least in part, mediated by FGFR1 activation and subsequent PPARγ activation-induced junction protein expression. Taken together with our previous investigations, rFGF21 might be developed as a novel and potent disease-modifying approach to treating ischemic stroke with T2DM.

## Abbreviations

T2DM	type 2 diabetes mellitus
BBB	blood—brain barrier
FGF21	fibroblast growth factor 21
rFGF21	recombinant fibroblast growth factor 21
FGFR1	fibroblast growth factor receptor 1
PPARγ	peroxisome proliferator-activated receptor gamma
dMCAO	distal middle cerebral artery occlusion
HBMEC	human brain microvascular endothelial cell
IL-1β	interleukin-1β
FITC	fluorescein isothiocyanate
EMSA	electrophoretic mobility shift assay
